# Ski Jumping Trajectory Reconstruction Using Wearable Sensors via Extended Rauch-Tung-Striebel Smoother with State Constraints

**DOI:** 10.3390/s20071995

**Published:** 2020-04-02

**Authors:** Xiang Fang, Benedikt Grüter, Patrick Piprek, Veronica Bessone, Johannes Petrat, Florian Holzapfel

**Affiliations:** 1 Institute of Flight System Dynamics, Technical University of Munich, 85748 Garching, Germany; benedikt.grueter@tum.de (B.G.); patrick.piprek@tum.de (P.P.); florian.holzapfel@tum.de (F.H.); 2 Department of Biomechanics in Sports, Faculty of Sport and Health Sciences, Technical University of Munich, 80992 Munich, Germany; veronica.bessone@tum.de (V.B.); johannes.petrat@tum.de (J.P.); 3 Olympic Training Center of Bavaria, 80809 Munich, Germany

**Keywords:** state estimation, constrained filtering, position and velocity estimation, inertial sensors, GPS, sensors fusion

## Abstract

To satisfy an increasing demand to reconstruct an athlete’s motion for performance analysis, this paper proposes a new method for reconstructing the position and velocity in the context of ski jumping trajectories. Therefore, state-of-the-art wearable sensors, including an inertial measurement unit, a magnetometer, and a GPS logger are used. The method employs an extended Rauch-Tung-Striebel smoother with state constraints to estimate state information offline from recorded raw measurements. In comparison to the classic inertial navigation system and GPS integration solution, the proposed method includes additional geometric shape information of the ski jumping hill, which are modeled as soft constraints and embedded into the estimation framework to improve the position and velocity estimation accuracy. Results for both simulated measurement data and real measurement data demonstrate the effectiveness of the proposed method. Moreover, a comparison between jump lengths obtained from the proposed method and video recordings shows the relative root-mean-square error of the reconstructed jump length is below 1.5 m depicting the accuracy of the algorithm.

## 1. Introduction

Acquiring an accurate estimation of the position and velocity of the athlete during a ski jump has always been of great interest to athletes, coaches, and sport researchers to analyze the movement for improving the jumping performance. Traditional solutions to meet this demand are video analysis techniques based on camera recorded videos [1]. However, such a system has the disadvantage that it generally only covers limited parts of the jumping area [2]. To record the entire jump, a large number of cameras would be required making the system expensive and hard to calibrate. Furthermore, camera systems merely provide information about the athlete’s position, while velocity and acceleration have to be estimated via numeric differentiation, which may be subject to large error.

In recent years, wearable sensors, such as inertial measurement units (IMUs) and global navigation satellite system (GNSS) receivers, have been widely employed for motion analysis in sports including ski jumping [3]. Chardonnens et al. [4] presented an IMU-based system measuring ski jumping dynamics including the position and velocity of the center of mass perpendicular to the table during take-off. Groh et al. [2] proposed a measurement system with IMUs and a light barrier to estimate the ski velocity and jump length. Blumenbach [5] designed and tested a helmet with a high-precision Global Positioning System (GPS) receiver for the positioning of ski jumpers. Fasel et al. [6] presented a solution to estimate the center of mass and its velocity using differential GNSS and IMUs for alpine skiing. In comparison to video analysis techniques, trajectory estimation using wearable IMUs and GNSS sensors has the advantages of being easy to use and maintain and having full coverage over all phases in a ski jump. For coaches and athletes, such a system provides the possibility to give feedback after each jump regarding the jumper’s performance, such as jump length and take-off velocity data.

Trajectory reconstruction techniques are a class of methods to achieve an accurate state estimation of a moving object by properly combining information from the kinematic model with measurement data from sensors, including but not limited to IMUs, magnetometers, and GNSS receivers. Trajectory reconstruction techniques are also termed flight path reconstruction in the aerospace field. A systematic overview of such methods for an aircraft can be found in [7]. Göttlicher and Holzapfel [8] reconstructed an aircraft’s flight path using low-cost sensors with the extended Rauch-Tung-Striebel (RTS) smoother. Similar techniques can be also applied to ski jumping applications.

From a methodological point of view, a trajectory reconstruction problem can be interpreted as a state estimation problem: One powerful tool to solve this is the classic RTS smoother, which was first proposed for linear systems in [9] using a linear Kalman filter [10] as the forward-time estimator. The extended RTS smoother is a modification of the classical RTS smoother capable of considering nonlinear systems. It employs the extended Kalman filter (EKF) as the forward-time filter to deal with the nonlinearity of the system. In this paper, the extended RTS smoother is adopted as an estimator. Moreover, we can formulate state equality constraints in a state estimation problem. Different methods to solve state estimation problems with state constraints, which are termed as constrained filtering (or smoothing) problems, are discussed in the survey [11].

In this paper, we present an offline method based on the extended RTS smoother to estimate the trajectory, including the position and velocity, of a ski jumper by the inertial navigation system and GPS integration. Additionally, to fully utilize all available information to improve the estimation quality, the geometric shape of the ski jumping hill is modeled as a set of additional soft constraints and included in the smoothing framework.

## 2. Data Acquisition

**Figure 1:**
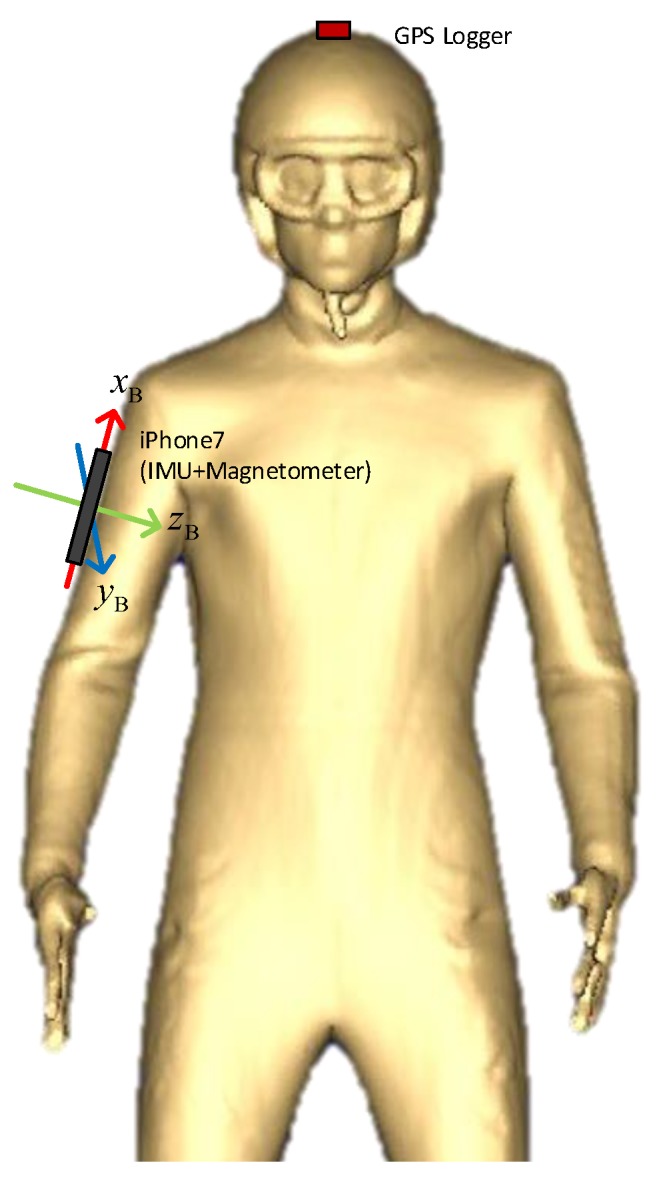
Illustrative figure on the placement of the sensors (based on a body scanning figure).

The measurement data were collected during a summer session in June 2018 on the jumping hill Schattenbergschanze (Hill Size = 106 m) in Oberstdorf, Germany. During this summer session, the hill slope was covered with water-soaked plastic. The measurement setup consists of the following sensors: a Qstarz BT Q1000eX GPS data logger and an iPhone 7, which contains an InvenSense ICM-20600 IMU, and an Alps HSCDTD008A magnetometer. Table 1 summarizes the performance characteristics of the sensors provided by the respective manufacturers. The GPS data logger provides position and velocity measurements with a 10 Hz sample rate. The IMU measures triaxial rotational rates as well as specific forces (i.e., non-gravitational accelerations) at 100 Hz frequency. The magnetometer provides triaxial local magnetic field flux density measurements also at 100 Hz. To the best of our knowledge, the IMU and magnetometer sensor chips in the iPhone 7 provide raw measurement data with a similar accuracy level as previous studies, e.g., [2,4,6,8]. The raw data measured by the smartphone was logged via the application Phyphox [12] and was attached to the jumper’s right upper arm using a running armband with the orientation shown in Figure 1. The GPS logger was fixed to the top of the helmet by adhesive tapes for better signal reception.

## 3. Methods

In this section, we first introduce the extended RTS smoother framework for a general system in Section 3.1. Next, modifications made for the joint state and parameter estimation as well as the state constraints in Section 3.2. Finally, the system model for ski jumping, which is implemented in the extended RTS smoother framework, is presented in Section 3.3.

### 3.1. Extended Rauch-Tung-Striebel Smoother

The extended RTS smoother is an offline estimation method utilizing the entire batch of measurements over a fixed time interval [16]. It combines a forward filtering pass using the EKF with a backward smoothing pass with the RTS smoother.

A state-space representation for a general nonlinear system described by ordinary differential equations (ODEs) can be written as

(1)x˙=f(x,u,w),x(t0)=x0,(2)y=g(x),(3)z=y+v,

where $x\in R^{n_{x}}$ is the state vector, $u\in R^{n_{u}}$ is the known input vector, $y\in R^{n_{y}}$ is the model outputs, and $z\in R^{n_{y}}$ represents the measurements. Symbols $w\in R^{n_{w}}$ and $v\in R^{n_{y}}$ denote process and measurement noise vectors. This system can be linearized and discretized into the following form [16]:

(4)xk=Φk−1xk−1+Γu,k−1uk−1+Γk−1wk−1,(5)yk=Hkxk,(6)zk=yk+vk,

where $H_{k}=\partial g(x_{k})/\partial x$. The transformation matrices $\Phi_{k-1}$, $\Gamma_{u,k-1}$, and $\Gamma_{k-1}$ are computed as

(7) $$\begin{array}{c} \Phi_{k-1}=\exp\left(A_{k-1}\Delta t_{k-1}\right)=\sum_{i=0}^{\infty }\frac{{\left(A_{k-1}\Delta t_{k-1}\right)}^{i}}{i!}\;, \end{array}$$

(8) $$\begin{array}{c} \Gamma_{u,k-1}=\int_{0}^{\Delta t_{k-1}}\exp\left(A_{k-1}\tau \right)B_{k-1}\;d\tau =\left(\sum_{i=1}^{\infty }\frac{A_{k-1}^{i-1}\Delta t_{k-1}^{i}}{i!}\right)B_{k-1}\;, \end{array}$$

(9) $$\begin{array}{c} \Gamma_{k-1}=\int_{0}^{\Delta t_{k-1}}\exp\left(A_{k-1}\tau \right)F_{k-1}\;d\tau =\left(\sum_{i=1}^{\infty }\frac{A_{k-1}^{i-1}\Delta t_{k-1}^{i}}{i!}\right)F_{k-1}\;, \end{array}$$

where $A_{k-1}=\frac{\partial f(x_{k-1},u_{k-1},w_{k-1})}{\partial x}$, $B_{k-1}=\frac{\partial f(x_{k-1},u_{k-1},w_{k-1})}{\partial u}$, $F_{k-1}=\frac{\partial f(x_{k-1},u_{k-1},w_{k-1})}{\partial w}$, and $\Delta t_{k-1}=t_{k}-t_{k-1}$. The noise processes $\left\{w_{k}\right\}$ and $\left\{v_{k}\right\}$ are assumed to be zero-mean, uncorrelated, white Gaussians, i.e., $w_{k}∼N\left(0,Q_{k}\right)$, $v_{k}∼N\left(0,R_{k}\right)$, $E\left[w_{j}w_{k}\right]=Q_{k}\delta_{jk}$, $E\left[v_{j}v_{k}\right]=R_{k}\delta_{jk}$, and $E[w_{j}v_{k}]=0$, where $\delta_{jk}$ is the Kronecker delta.

#### 3.1.1. Extended Kalman Filter

The EKF is a sequential state estimation method for nonlinear systems [11,16]. First, the initial state ${\hat{x}}_{0|0}$ and covariance $P_{0|0}$ for the EKF are given as

(10) $$\begin{array}{cc} {\hat{x}}_{0|0} & ={\bar{x}}_{0}\;, \end{array}$$

(11) $$\begin{array}{cc} P_{0|0} & ={\bar{P}}_{0}\;, \end{array}$$

where ${\bar{x}}_{0}$ is an a priori estimate of the initial states $x_{0}$ and ${\bar{P}}_{0}$ is the covariance of the estimation error of ${\bar{x}}_{0}$. Then, for each time point $t_{k}$ with k∈{1,2,…,N}, the EKF consist of a prediction and an update step as follows:

1) Prediction step:

(12) $$\begin{array}{c} {\hat{x}}_{k|k-1}={\hat{x}}_{k-1|k-1}+\int_{t_{k-1}}^{t_{k}}f\left({\hat{x}}_{k-1|k-1},{\tilde{u}}_{k-1},0\right)\;d\tau \;, \end{array}$$

(13) $$\begin{array}{c} P_{k|k-1}=\Phi_{k-1}^{⊤}P_{k-1|k-1}\Phi_{k-1}+\Gamma_{k-1}^{⊤}Q_{k-1}\Gamma_{k-1}\;, \end{array}$$

where ${\hat{x}}_{k|k-1}$ represents the a priori state estimate at time $t_{k}$ based on measurements up to time $t_{k-1}\;$, i.e., ${\hat{x}}_{k|k-1}=E\left(x_{k}|z_{1},\;\cdots ,z_{k-1}\right)$, and $P_{k|k-1}$ denotes the covariance of the estimation error of ${\hat{x}}_{k|k-1}\;$. To evaluate $\Phi_{k-1}$ and $\Gamma_{k-1}$, matrices $A_{k-1}$ and $F_{k-1}$ in Equation (7) and Equation (9) are linearized as $A_{k-1}=\frac{\partial f({\hat{x}}_{k-1|k-1},u_{k-1},0)}{\partial x}$ and $F_{k-1}=\frac{\partial f({\hat{x}}_{k-1|k-1},u_{k-1},0)}{\partial w}$. The integral in Equation (12) is numerically solved via the classic 4-th order Runge–Kutta method with ${\tilde{u}}_{k-1}=\frac{t_{k}-\tau }{t_{k}-t_{k-1}}u_{k-1}+\frac{\tau -t_{k-1}}{t_{k}-t_{k-1}}u_{k}$.

2) Update step:

(14) $$\begin{array}{c} K_{k}=P_{k|k-1}H_{k}^{⊤}{\left[H_{k}P_{k|k-1}H_{k}^{⊤}+R_{k}\right]}^{-1}\;, \end{array}$$

(15) $$\begin{array}{c} {\hat{x}}_{k|k}={\hat{x}}_{k|k-1}+K_{k}\left[z_{k}-g\left({\hat{x}}_{k|k-1}\right)\right]\;, \end{array}$$

(16) $$\begin{array}{c} P_{k|k}=\left[I_{n_{x}}-K_{k}H_{k}\right]P_{k|k-1}\;, \end{array}$$

where ${\hat{x}}_{k|k}$ denotes the a posteriori state estimate at time $t_{k}$ based on measurements up to time $t_{k}$, i.e., ${\hat{x}}_{k|k}=E\left(x_{k}|z_{1},\;\cdots ,z_{k}\right)$, and $P_{k|k}$ denotes the covariance of the estimation error of ${\hat{x}}_{k|k}$. The symbol $I_{n_{x}}$ denotes an $n_{x}$ by $n_{x}$ identity matrix, and $H_{k}$ is approximated by $H_{k}=\partial g({\hat{x}}_{k|k-1})/\partial x$.

#### 3.1.2. Rauch-Tung-Striebel Smoother

After the forward-time filtering pass, the EKF estimation of the states ${\hat{x}}_{N|N}$ and its error covariance $P_{N|N}$ is used to initialize the corresponding values in the RTS smoother for the final time point $t_{N}$:

(17) $$\begin{array}{cc} {\hat{x}}_{N}^{s} & ={\hat{x}}_{N|N}\;, \end{array}$$

(18) $$\begin{array}{cc} P_{N}^{s} & =P_{N|N}\;, \end{array}$$

where the superscripts “*s*” indicate the smoothed estimates. Then, the RTS smoother runs backward in time for $t_{k}$ with k∈{N−1,N−2,…,0} as

(19) $$\begin{array}{c} K_{k}^{s}=P_{k|k}\Phi_{k}^{⊤}{\left(P_{k+1|k}\right)}^{-1}\;, \end{array}$$

(20) $$\begin{array}{c} {\hat{x}}_{k}^{s}={\hat{x}}_{k|k}+K_{k}^{s}\left[{\hat{x}}_{k+1}^{s}-{\hat{x}}_{k+1|k}\right]\;, \end{array}$$

(21) $$\begin{array}{c} P_{k}^{s}=P_{k|k}+K_{k}^{s}\left[P_{k+1}^{s}-P_{k+1|k}\right]{\left(K_{k}^{s}\right)}^{⊤}\;, \end{array}$$

where the smoothed estimate ${\hat{x}}_{k}^{s}$ can be regarded as the state estimate of time point $t_{k}$ utilizing all measurements, i.e., ${\hat{x}}_{k}^{s}=E\left(x_{k}|z_{1},\;\ldots ,z_{N}\right)$ [17], and $P_{k}^{s}$ is the covariance of the estimation error of ${\hat{x}}_{k}^{s}$.

### 3.2. Adaption for Parameter Estimation and Soft Constraints

To account for unknown parameters and known constraints for the estimation problem, the extended RTS smoother for state estimation is modified by the following adaptations.

#### 3.2.1. Joint State and Parameter Estimation

When the model also includes parameters $p\in R^{n_{p}}$ to be estimated, these parameters are treated as additional states with $\dot{p}=0$ so that they are compatible with the extended RTS smoother framework. Therefore, with the augmented state vector defined as $x_{a}={\left[x^{⊤}\;p^{⊤}\right]}^{⊤}\in R^{n_{x}+n_{p}}$, the augmented system ODEs become

(22) $${\dot{x}}_{a}=f_{a}\left(x_{a},u,w\right)=\left[\begin{array}{c} f(x,u,p,w)\\ 0 \end{array}\right]\;.$$

#### 3.2.2. Constrained Filtering

Suppose that a system satisfies the nonlinear state equality constraints as

(23) $$c_{k}=h\left(x_{k}\right)\;.$$

In order to include these into the extended RTS smoother framework, the constraints can be treated as additional pseudo measurements in the form of

(24) $$\left[\begin{array}{c} z_{k}\\ c_{k} \end{array}\right]=\left[\begin{array}{c} y_{k}\\ h(x_{k}) \end{array}\right]+\left[\begin{array}{c} v_{k}\\ v_{c,k} \end{array}\right]\;.$$

For the case that equality constraints are satisfied exactly, which is termed as hard constraints in [11,18], the pseudo measurements can be treated as perfect with pseudo measurement noises $v_{c,k}=0$. Contrary, soft constraints are only required to be fulfilled approximately, i.e., $c_{k}\approx h\left(x_{k}\right)$. In this case, we assume $v_{c,k}$ are zero-mean Gaussian noises with small covariance $Q_{c}$, i.e., $v_{c,k}∼N\left(0,Q_{c}\right)$.

We further write Equation (24) as

(25) $$\begin{array}{cc} y_{a} & =g_{a}\left(x_{a}\right)\;, \end{array}$$

(26) $$\begin{array}{cc} z_{a} & =y_{a}+v_{a}\;, \end{array}$$

where $z_{a}={\left[z_{k}^{⊤}\;c_{k}^{⊤}\right]}^{⊤}$, $y_{a}={\left[y_{k}^{⊤}\;h{\left(x_{k}\right)}^{⊤}\right]}^{⊤}$, and $v_{a}={\left[v_{k}^{⊤}\;v_{c,k}^{⊤}\right]}^{⊤}$. By Equations (22), (25), and (26), we formulated the state-space presentation of an augmented system for state and parameter estimation with state constraints in the form of Equations (1)–(3).

### 3.3. System Model

After introducing the estimation framework for a general system in the previous two subsections, the system model for the ski jumping application is presented in this subsection.

#### 3.3.1. Coordinate Frame Definitions

**Figure 2:**
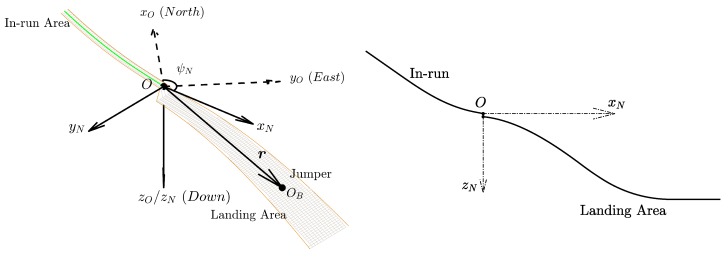
Definitions of the local north-east-down frame and the hill reference frame.

**Figure 3:**
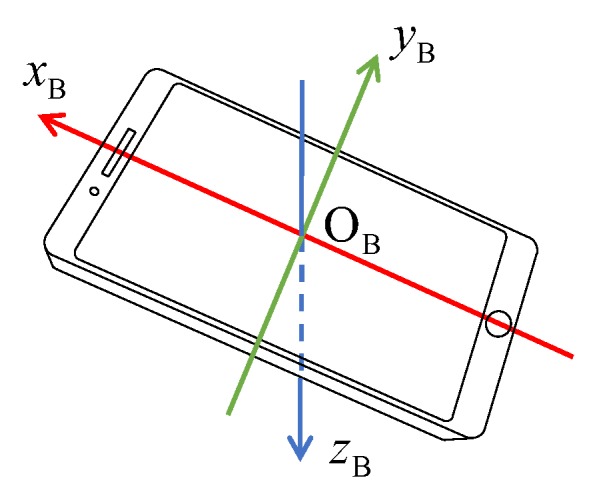
Definition of the sensor body frame.

The first necessary frame is the hill reference frame $Ox_{N}y_{N}z_{N}$. As shown in Figure 2, its origin O is located at the end of the in-run table. The $x_{N}$ axis and $z_{N}$ axis are both within the symmetric plane of the jumping hill and point horizontally forward and vertically down, respectively. The $y_{N}$ axis is perpendicular to $x_{N}$ and $z_{N}$ axes to form a right-handed coordinate system. $r_{N}={\left[x_{N}\;y_{N}\;z_{N}\right]}^{⊤}\;$ denotes the coordinate matrix in the hill reference frame of the jumper’s (or, to be exact, the IMU sensor’s) relative position vector r with respect to the origin O. Another necessary frame is the local north-east-down (NED) frame $Ox_{O}y_{O}z_{O}$. The origin of the local NED frame coincides with point O, and the three axes $x_{O}$, $y_{O}$ and $z_{O}$ point to north, east, and down directions respectively. Finally, the sensor’s body frame $O_{B}x_{B}y_{B}z_{B}$ is defined as following: the origin $O_{B}$ is located at the center of the cellphone, and the three axes are fixed to its three axes of symmetry as shown in Figure 3. The coordinates in the NED frame of the relative position vector r are denote by $r_{O}={\left[x_{O}\;y_{O}\;z_{O}\right]}^{⊤}\;$.

#### 3.3.2. Conversion of GPS Position Measurements to the Hill Reference Frame

The position measurements from GPS receivers are usually provided in geodetic coordinates, such as the World Geodetic System 1984 (WGS84) frame [19], as a time sequence of latitude μ, longitude λ, and height *h*. For ski jumping applications, it is more useful to calculate relative positions of the ski jumper with respect to the jumping hill.

With the frame definitions stated before, the GPS-measured positions $r_{\mathrm{WGS},\mathrm{GPS}}={\left[\mu \;\lambda \;h\right]}^{⊤}\;$ in the WGS84 frame are first transformed into the local NED frame $r_{O,\;\mathrm{GPS}}={\left[x_{O}\;y_{O}\;z_{O}\right]}^{⊤}\;$ by the following equations:

(27)xO=(Mμ+h)(μ−μ0),(28)yO=(Nμ+h)cosμ0(λ−λ0),(29)zO=−(h−h0),

where $\mu_{0}$, $\lambda_{0}$, and $h_{0}$ are the latitude, longitude, as well as height of the hill reference frame’s origin O, and parameters for the WGS84 reference ellipsoid [19] can be found in Table 2.

Then, the GPS-measured positions $r_{N,\;\mathrm{GPS}}$ in the hill reference frame are obtained by

(30) $$r_{N,\;\mathrm{GPS}}=M_{\mathrm{NO}}r_{O,\;\mathrm{GPS}}\;,$$

where $M_{\mathrm{NO}}$ is the transformation matrix from local NED to hill reference frame as

(31) $$M_{\mathrm{NO}}=\left[\begin{array}{ccc} \;\;\;\cos\psi_{N} & \sin\psi_{N} & 0\\ -\sin\psi_{N} & \cos\psi_{N} & 0\\ 0 & 0 & 1 \end{array}\right]\;,$$

where $\psi_{N}$ denotes the hill azimuth angle, which is defined as the rotation angle from the $x_{O}$ axis (the north) to the $x_{N}$ axis, as shown in Figure 2.

#### 3.3.3. Constraint Modeling of the Geometric Shape of a Ski Jumping Hill

The geometric shape of the ski jumping hill is used as additional a priori information and included in the trajectory reconstruction problem as a set of constraints for further improvement of the estimation quality. The mathematical model of a ski jumping hill is described in [20], which was published by the International Ski Federation (Fédération Internationale de Ski, FIS) as the guideline to construct ski jumping hills. Additionally, all necessary model parameters can be found in the jumping hill certificate, which is also issued by the FIS. In our case, we have the certificate of jumping hill - No. 215/GER 29 [21] for the Schattenbergschanze.

**Figure 4:**
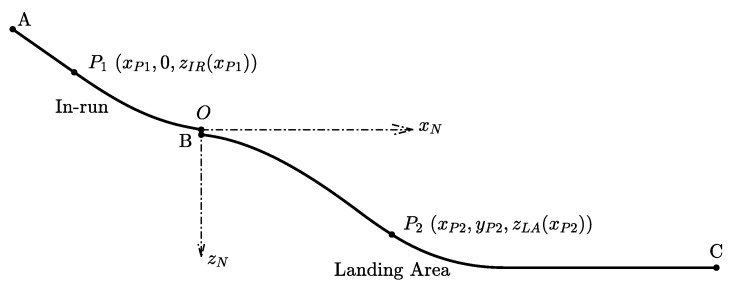
The Longitudinal Profile of a Ski Jumping Hill.

Figure 4 shows the geometric profile of a FIS-certified ski jumping hill in the longitudinal plane. The curve from the point A to the origin O is called the in-run where the jumper skis down following the track before take-off. For any point $P_{1}$ on the in-run curve, we assume its coordinates to be $\left(x_{P1},\;0,\;z_{\mathrm{IR}}\left(x_{P1}\right)\right)$ where $z_{P1}=z_{\mathrm{IR}}\left(x_{P1}\right)$ denotes the mapping relationship for the in-run in the vertical plane. Since the trajectory of the jumper before take-off must coincide with the in-run curve, we define the constraints for the in-run curve in the longitudinal and lateral plane as

(32) $$\begin{array}{c} c_{V,\;\mathrm{IR}}=z_{N}-z_{\mathrm{IR}}\left(x_{N}\right)\;, \end{array}$$

(33) $$\begin{array}{c} c_{H,\;\mathrm{IR}}=y_{N}\;. \end{array}$$

When the jumper is in the in-run area before take-off, i.e., $t_{k}\leq t_{TO}$, the constraints should fulfill that $c_{V,\;\mathrm{IR}}\approx 0$ and $c_{H,\;\mathrm{IR}}\approx 0$. The curve BC in Figure 4 is the landing area (including the landing slope and the out-run). For a point in the landing area $P_{2}$, its position coordinate in the hill reference frame is $\left(x_{P2},\;y_{P2},\;z_{\mathrm{LA}}\left(x_{P2}\right)\right)$ where $z_{P2}=z_{\mathrm{LA}}\left(x_{P2}\right)$ is the mapping relationship of the landing area in vertical plane. The jumper would land somewhere in between and afterward skiing along this surface. Therefore, we can formulate another constraint as follows:

(34) $$c_{V,\;\mathrm{LA}}=z_{N}-z_{\mathrm{LA}}\left(x_{N}\right)\;.$$

After the jumper’s landing, i.e., $t_{k}\geq t_{TD}\;$, the constraints should be active such that $c_{V,\;\mathrm{LA}}\approx 0\;$. The method for detecting the take-off time point $t_{TO}$ and the touch-down time point $t_{TD}$ using raw measurements is introduced in Appendix A.

#### 3.3.4. Measurement Error Models

To model the measurement error of the inertial sensor, constant bias terms Δω and Δa for the gyroscope and accelerometer as well as zero-mean, white, Gaussian noise terms $w_{\mathrm{gyro}}$ and $w_{\mathrm{acc}}$ are explicitly considered as

(35) $$\begin{array}{cc} \omega_{B,\;\mathrm{gyro}} & =\omega_{B}+\Delta \omega +w_{\mathrm{gyro}}\;, \end{array}$$

(36) $$\begin{array}{cc} a_{B,\;\mathrm{acc}} & =a_{B}+\Delta a+w_{\mathrm{acc}}\;, \end{array}$$

where $\omega_{B,\;\mathrm{gyro}}$ represents the rotational rates measurement from the gyroscope, $a_{B,\;\mathrm{acc}}$ denotes the measurement from the accelerometer, and $\omega_{B}$ as well as $a_{B}$ are the true rotational rates and specific forces respectively.

The measurement error model of the magnetometer is considered to be

(37) $$m_{B,\;\mathrm{mag}}=\left(1+\mathrm{diag}\left(\Delta S_{m}\right)\right)m_{B}+\Delta m+v_{\mathrm{mag}}\;,$$

where $m_{B,\;\mathrm{mag}}$ is the magnetometer measurement of the local magnetic field strength, $\Delta S_{m}$ represents the vector of the scaling factor error and $\mathrm{diag}(\Delta S_{m})\;$ denotes the corresponding square matrix with $S_{m}$ being its diagonal elements, Δm denotes the constant bias vector, $m_{B}$ is the true local magnetic field strength, and $v_{\mathrm{mag}}$ represents the zero-mean, white, Gaussian measurement noise.

The GPS measurements are modeled as

(38) $$\begin{array}{cc} r_{N,\mathrm{GPS}} & =r_{N}+v_{\mathrm{pos}}\;, \end{array}$$

(39) $$\begin{array}{cc} v_{O,\mathrm{GPS}} & =v_{O}+v_{\mathrm{vel}}\;, \end{array}$$

where $r_{N}$ is the true position in the hill reference frame, $v_{O}$ is the true velocity in the local NED frame, $r_{N,\mathrm{GPS}}$ and $v_{O,\mathrm{GPS}}$ are the GPS measurements accordingly, and $v_{\mathrm{pos}}$ and $v_{\mathrm{vel}}$ are the measurement noise terms.

#### 3.3.5. State, Output, and Constraint Equations

The system states vector x is given by

(40) $$x={\left[\begin{array}{ccc} r_{N}^{⊤} & v_{B}^{⊤} & q_{\mathrm{BO}}^{⊤} \end{array}\right]}^{⊤}\;,$$

where $v_{B}$ denotes the triaxial velocity in the body-fixed frame, and $q_{\mathrm{BO}}$ represents the attitude quaternions of the body-fixed frame with respect to the local NED frame. The input vector u contains the IMU measurements $\omega_{B,\;\mathrm{gyro}}$ and $a_{B,\;\mathrm{acc}}$ as

(41) $$u={\left[\begin{array}{cc} \omega_{B,\;\mathrm{gyro}}^{⊤} & a_{B,\;\mathrm{acc}}^{⊤} \end{array}\right]}^{⊤}\;.$$

The parameter vector p to be estimated includes the biases of the gyroscope Δω, the accelerometer Δa, the magnetometer Δm, and the scaling factor error of the magnetometer $\Delta S_{m}$:

(42) $$p={\left[\begin{array}{cccc} \Delta \omega^{⊤} & \Delta a^{⊤} & \Delta m^{⊤} & \Delta S_{m}^{⊤} \end{array}\right]}^{⊤}.$$

The process noise vector w consists of influences from both gyroscope and accelerometer:

(43) $$w={\left[\begin{array}{cc} w_{B,\;\mathrm{gyro}}^{⊤} & w_{B,\;\mathrm{acc}}^{⊤} \end{array}\right]}^{⊤}\;.$$

With the definitions above, the state equations $\dot{x}=f\left(x,u,p,w\right)$ stated in Equation (22) can be assembled by the equations of inertial navigation mechanism and kinematics as follows:

(44) $$\begin{array}{cc} {\dot{r}}_{N} & =M_{\mathrm{NO}}M_{\mathrm{BO}}^{⊤}v_{B}\;, \end{array}$$

(45) $$\begin{array}{cc} {\dot{v}}_{B} & =a_{B}+M_{\mathrm{BO}}g_{O}+\omega_{B}\times v_{B}\;, \end{array}$$

(46) $$\begin{array}{cc} {\dot{q}}_{\mathrm{BO}} & =\frac{1}{2}\left[\begin{array}{rrr} -q_{1} & -q_{2} & -q_{3}\\ q_{0} & -q_{3} & q_{2}\\ q_{3} & q_{0} & -q_{1}\\ -q_{2} & q_{1} & q_{0} \end{array}\right]\omega_{B},\; \end{array}$$

where $M_{\mathrm{BO}}$ is the transformation matrix from the local NED frame to the body-fixed frame as

(47) $$M_{\mathrm{BO}}=\left[\begin{array}{ccc} q_{0}^{2}+q_{1}^{2}-q_{2}^{2}-q_{3}^{2} & 2\left(q_{1}q_{2}+q_{0}q_{3}\right) & 2\left(q_{1}q_{3}-q_{0}q_{2}\right)\\ 2\left(q_{1}q_{2}-q_{0}q_{3}\right) & q_{0}^{2}-q_{1}^{2}+q_{2}^{2}-q_{3}^{2} & 2\left(q_{2}q_{3}+q_{0}q_{1}\right)\\ 2\left(q_{0}q_{2}+q_{1}q_{3}\right) & 2\left(q_{2}q_{3}-q_{0}q_{1}\right) & q_{0}^{2}-q_{1}^{2}-q_{2}^{2}+q_{3}^{2} \end{array}\right],$$

and $g_{O}={\left[\begin{array}{ccc} 0 & 0 & g_{0} \end{array}\right]}^{⊤}$ represents the gravitational vector in the local NED frame. The term $a_{B}$ in Equation (45) can be substituted by $a_{B}=a_{B,\;\mathrm{acc}}-\Delta a-w_{\mathrm{acc}}$ using the accelerometer measurement model Equation (36). Similarly, $\omega_{B}$ can be written as $\omega_{B}=\omega_{B,\;\mathrm{gyro}}-\Delta \omega -w_{\mathrm{gyro}}$ via the gyroscope measurement model Equation (35) and be further substituted into Equation (46).

The output vector y is written as

(48) $$y={\left[\begin{array}{ccc} r_{N}^{⊤} & v_{O}^{⊤} & m_{B,\;\mathrm{meas}}^{⊤} \end{array}\right]}^{⊤}\;,$$

where $r_{N}$ can be directly obtained from the state vector, the velocity $v_{O}$ is calculated as

(49) $$v_{O}=M_{\mathrm{BO}}^{⊤}v_{B}\;,$$

and $m_{B,\;\mathrm{meas}}\;$ is the estimated magnetometer measurement. By taking the magnetometer model Equation (37) into account, $m_{B,\;\mathrm{meas}}\;$ is estimated by

(50) $$m_{B,\;\mathrm{meas}}=\left(1+\mathrm{diag}\left(\Delta S_{m}\right)\right)M_{\mathrm{BO}}m_{O,\;\mathrm{WMM}}+\Delta m\;,$$

where $m_{O,\;\mathrm{WMM}}$ is the reference value of the local magnetic field strength in the local NED frame from the World Magnetic Model 2015 [22].

Accordingly, the measurement vector is $z={\left[\begin{array}{ccc} r_{N,\;\mathrm{GPS}}^{⊤} & v_{O,\;\mathrm{GPS}}^{⊤} & m_{B,\;\mathrm{mag}}^{⊤} \end{array}\right]}^{⊤}$ with measurements from the GPS logger ($r_{N,\;\mathrm{GPS}}$ and $v_{O,\;\mathrm{GPS}}$) and the magnetometer ($m_{B,\;\mathrm{mag}}$). The measurement noise vector is therefore $v={\left[\begin{array}{ccc} v_{\mathrm{pos}}^{⊤} & v_{\mathrm{vel}}^{⊤} & v_{\mathrm{mag}}^{⊤} \end{array}\right]}^{⊤}\;$.

The vector of constraints c is defined to be

(51) $$c={\left[\begin{array}{cccc} c_{q} & c_{V,\;\mathrm{IR}} & c_{H,\;\mathrm{IR}} & c_{V,\;\mathrm{LA}} \end{array}\right]}^{⊤},$$

where $c_{q}={∥q_{\mathrm{BO}}∥}_{2}-1$ is the constraint for attitude quaternions. Here, $c_{q}$ ensures a unit quaternion, i.e., ${∥q_{\mathrm{BO}}∥}_{2}=1$. The constraints $c_{V,\;\mathrm{IR}}$, $c_{H,\;\mathrm{IR}}$, and $c_{V,\;\mathrm{LA}}$ are geometric constraints given by the shape of the jumping hill, which are introduced in Section 3.3.3.

## 4. Results and Discussion

In this Section, trajectory reconstruction results from both simulated and real measurement data are presented. The configuration for the extended RTS smoother is first introduced in Section 4.1. The simulated measurements are used to theoretically validate the purposed method in Section 4.2. Results based on real measurements are presented in Section 4.3. The jump length obtained from both the proposed method and video recordings are compared in Section 4.4 to further validate the results based on real measurement data.

### 4.1. Setting

We first introduce necessary configurations for the extended RTS smoother algorithm. For the initial states ${\bar{x}}_{0}={\left[\begin{array}{ccc} {\bar{r}}_{N,0}^{⊤} & {\bar{v}}_{O,0}^{⊤} & {\bar{q}}_{0} \end{array}\right]}^{⊤}\;$, the GPS measurement at initial time $t_{0}$ is adopted as an estimation for the initial position and velocity as ${\bar{r}}_{N,0}=r_{N,\;\mathrm{GPS},0}$ and ${\bar{v}}_{O,0}=v_{O,\;\mathrm{GPS},0}$. The estimated value for initial attitude ${\bar{q}}_{0}\;$ is calculated via the factored quaternion algorithm proposed in [23] using the accelerometer and magnetometer measurements at time $t_{0}$. The initial guess for all measurement error parameters is chosen to be zero as ${\bar{p}}_{0}={\left[\begin{array}{cccc} \Delta \omega_{0}^{⊤} & \Delta a_{0}^{⊤} & \Delta m_{0}^{⊤} & \Delta S_{m,0}^{⊤} \end{array}\right]}^{⊤}=0\;$. For the augmented initial states ${\bar{x}}_{a,0}={\left[\begin{array}{cc} {\bar{x}}_{0}^{⊤} & {\bar{p}}_{0}^{⊤} \end{array}\right]}^{⊤}$, the estimated covariance matrix for the initial states error ${\bar{P}}_{0}$ is set as shown in Table 3. In addition, the covariance matrix of noises Q and R is considered constant and the estimated values of Q and R are listed in Table 4 and Table 5 respectively. Within the model, the hill azimuth angle $\psi_{N}$ is set as 312.6° for the jumping hill Schattenbergschanze. The gravitational constant at the site is calculated to be $g_{0}=9.8053\;m/s^{2}$ according to the Earth Gravitational Model 1996 [19].

### 4.2. Validation by Simulated Measurement Data

To validate the accuracy of the proposed algorithm, an artificial measurement data set is generated by assuming a true value and adding sensor error and noise. This data set is generated based on the result from real measurements as a reference trajectory, ensuring similar observability of the estimation problem as in the real world. Trajectory optimization techniques with least-squares costs are used to obtain a trajectory with similar acceleration and angular velocities as the measured data. By adding path constraints to the trajectory optimization, the true trajectory also fulfills the constraints in Equations (32)–(34). The true value for the generated trajectory also complies with the kinematic models in Equations (44)–(46). The sensor errors and noises are added to the true values according to Equations (35)–(39), with the noise covariance values shown in Table 4 and Table 5 to generate artificial measurements.

**Figure 5:**
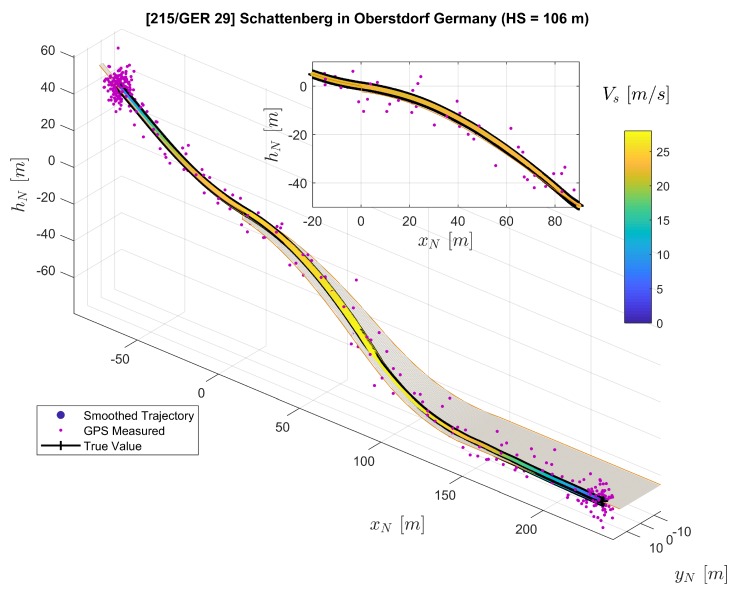
Comparison of the extended RTS smoother reconstructed trajectory (color-coded line), the generated reference trajectory (black line), and the GPS measured trajectory (purple dots) plotted on the ski jumping hill model.

**Figure 6:**
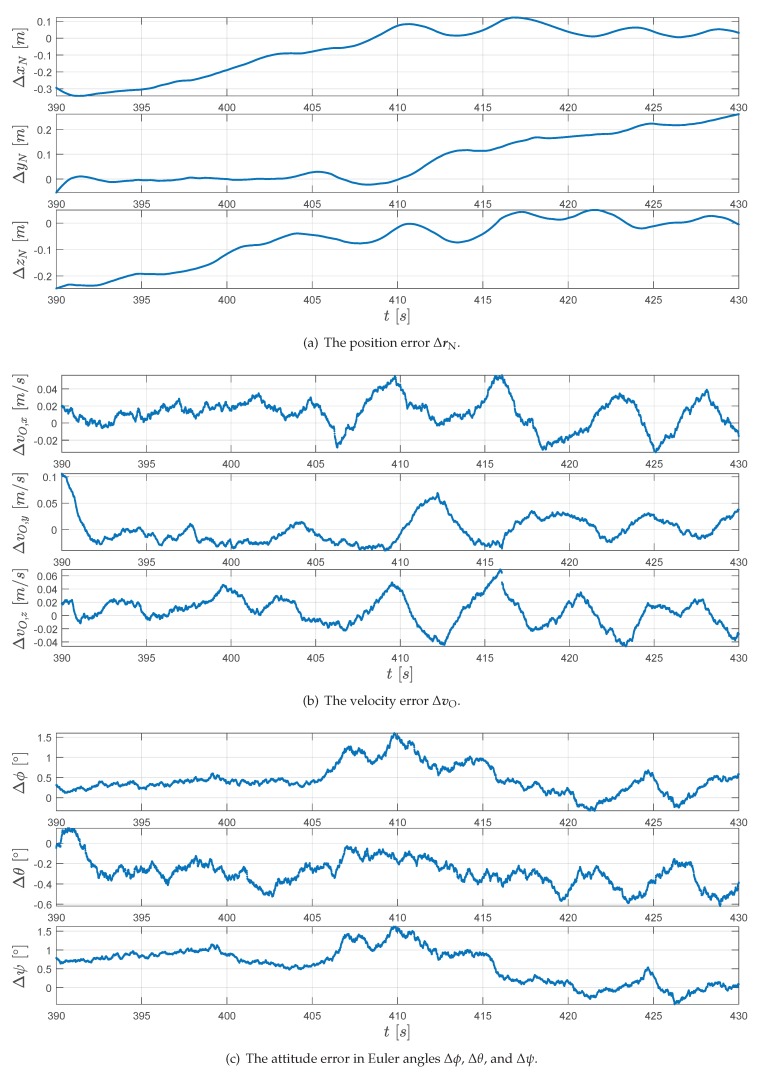
The RTS smoother estimation error for the generated reference data.

The trajectory is reconstructed with only the noisy artificial measurements known to the extended RTS smoother. The reconstruction result is presented in Figure 5. Here, it can be observed that the smoothed trajectory agrees closely with the true reference. To quantify the error of the estimated position and velocity, we calculated the error by

(52) $$\begin{array}{c} \Delta r_{N}={\hat{r}}_{N}^{s}-r_{N,\mathrm{ref}}\;, \end{array}$$

(53) $$\begin{array}{c} \Delta v_{O}={\hat{v}}_{O}^{s}-v_{O,\mathrm{ref}}\;, \end{array}$$

where variables with subscript “ref” are the true values from the generated data. The error of these estimated positions and velocities are presented in Figure 6a,b respectively. To intuitively display the attitude, the quaternions $q_{\mathrm{BO}}$ are converted to the corresponding Euler angles (in z-y-x order): the bank angle ϕ, the pitch angle θ, and the azimuth angle ψ by

(54)ϕ=arctan2q2q3+q0q1q02−q12−q22+q32,(55)θ=arcsin−2q1q3−q0q2,(56)ψ=arctan2q1q2+q0q3q02+q12−q22−q32.

Then, we use the error in Euler angles to represent the attitude estimation error as

(57) $$\begin{array}{c} \Delta \phi ={\hat{\phi }}^{s}-\phi_{\mathrm{ref}}\;,\;\Delta \theta ={\hat{\theta }}^{s}-\theta_{\mathrm{ref}}\;,\;\mathrm{and}\;\Delta \psi ={\hat{\psi }}^{s}-\psi_{\mathrm{ref}}\;. \end{array}$$

The Euler angle estimation error is presented in Figure 6c. The root-mean-square (RMS) error for position, velocities, and Euler angles are summarized in Table 6. This result shows that the proposed algorithm can successfully reconstruct the trajectory and that the position accuracy for the assumed noise covariance is in decimeter magnitudes.

### 4.3. Validation by Real Measurement Data

Now, we apply the extended RTS smoother on real measurement data to the ski jumping trajectory reconstruction problem. The results for a reconstructed ski jump are presented in the following part.

**Figure 7:**
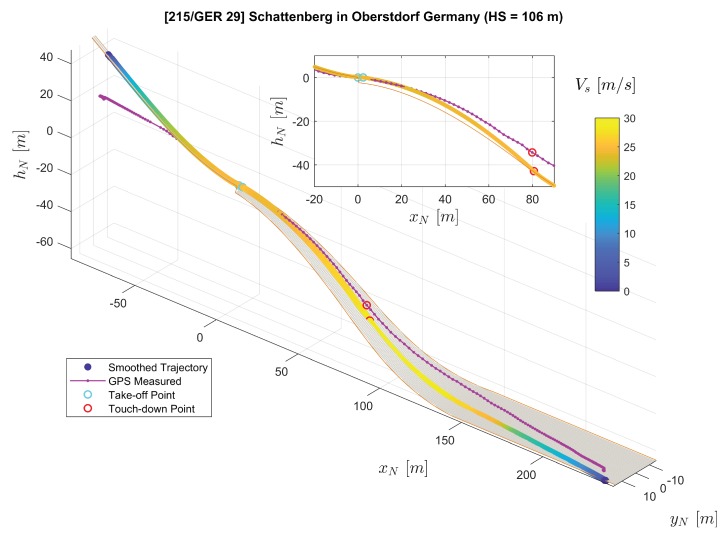
Comparison of the extended RTS smoother reconstructed trajectory (color-coded line) and the GPS measured trajectory (purple solid line with dots) plotted on the ski jumping hill model (orange lines and the gray surface).

**Figure 8:**
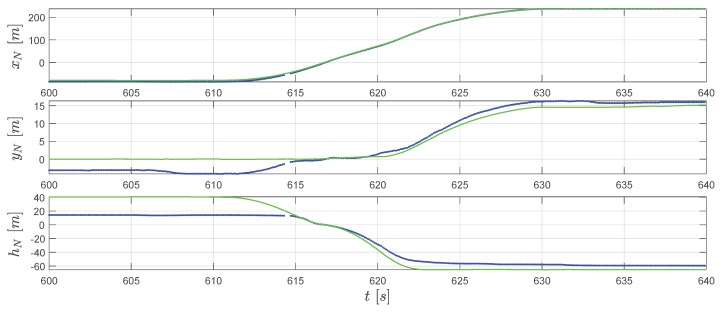
Converted GPS position measurements $r_{N,\;\mathrm{GPS}}$ (blue dots) and the extended RTS smoother reconstructed relative positions $r_{N}$ (green line).

**Figure 9:**
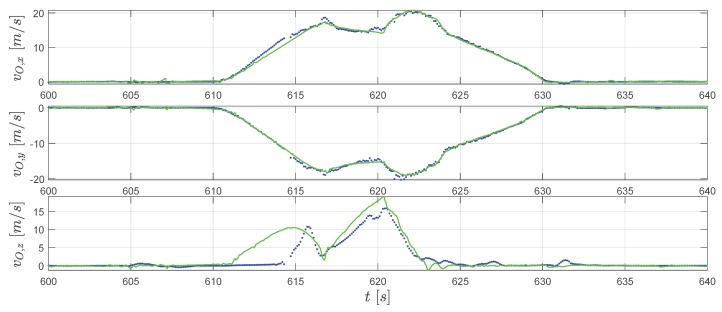
GPS velocity measurements $v_{O,\;\mathrm{GPS}}$ (blue dots) and the extended RTS smoother reconstructed velocity $v_{O}$ (green line).

In Figure 7, the three-dimensional trajectories of the jumper are plotted with a digital ski jumping hill model based on [20,21]. The purple solid line with dots shows the converted raw GPS position measurements $r_{N,\;\mathrm{GPS}}$ where each dot on the line represents a measurement sample. Furthermore, the line consisting of color-coded dots represents position reconstructed by the extended RTS smoother, where the color of one dot represent the velocity $V_{s}={∥v_{O}∥}_{2}=\sqrt{v_{O,\;x}^{2}+v_{O,\;y}^{2}+v_{O,\;z}^{2}}$. Note that we use the relative local height $h_{N}$ here instead of $z_{N}$ in the figure for convenience. The relationship between $h_{N}$ and $z_{N}$ is directly $h_{N}=-z_{N}$. The subplot on the top-right corner of Figure 7 presents the projection of the jumping trajectories on the $x_{N}$-$z_{N}$ plane (focusing mainly on the flight part). The comparison between GPS-measured and extended RTS smoother reconstructed position as well as velocity time histories is presented in Figure 8 and Figure 9 respectively.

Both GPS measurements and the smoother estimation in Figure 7 show the trajectory of an entire jump from start to stop. By comparing the GPS-measured trajectory to the jumping hill model for the in-run and out-run parts, it shows that the GPS height channel contains relatively large errors. After the jumper starts his run along the track during the in-run phase, the GPS-measured trajectory indicates that the jumper starts to move horizontally, but remains at the same height vertically. For part after the touch-down in the landing area, the GPS-measured trajectory shows large error leading to the position being above the ground. Therefore, in this case, the GPS-measured trajectory is not adequate for the demand for analyzing the ski jump limited to its accuracy. It is worth mentioning that the GPS measured trajectory does not necessarily encounter the same type of error in the height channel as in our case. Nevertheless, due to the trilateration positioning principle of the GPS technology employed, the GPS height measurement is far less accurate than the latitude and longitude measurements. The GPS service standard [24] reported that the GPS has a global average positioning accuracy of ≤9m(95%) horizontal error and ≤15m(95%) vertical error.

On the other hand, besides the GPS measurements as a data source, the extended RTS smoother uses IMU and magnetic measurements as well as geometric information of the jumping hill to perform data fusion. The application of the RTS smoother reconstructs the trajectory despite a lower GPS measurement frequency and the existence of some GPS height errors as shown in Figure 7, Figure 8 and Figure 9. In Figure 7, the reconstructed trajectory shows the entire process of the jump. The jumper first slides down along the in-run track with increasing speed $V_{S}$. For the flight part, this can be clearly observed from both the three-dimensional plot and the subplot in Figure 7. It can be observed that the height above the ground is decreasing until the jumper’s touch-down with the speed further increasing. After the touch-down, the jumper skis down on the landing area of the hill with his speed still accelerating. After reaching the flat part at the hill bottom, the jumper decelerates due to the friction and his break action and then comes to a full stop. In comparison to the GPS measurement, the estimated position and velocity are improved especially in the height channel, which reveals more information for the jump analysis, and the sampling frequency on the trajectory is also increased.

In Figure 8, we can observe that two lines fit well for $x_{N}$. For $y_{N}$ in the extended RTS smoother result (the green line), the constraint $c_{H,\;\mathrm{IR}}$ correct the position $y_{N}$ to 0 before take-off. Afterward, the reconstructed trajectory matches the GPS measurements with some differences. For the $h_{N}$ channel, as also mentioned before, the raw GPS measurement has a relatively large error which can be clearly observed in Figure 7. The smoother reconstructed height by considering the vertical constraints $c_{V,IR}$ and $c_{V,LA}$ is moving in the in-run curve before the take-off and on the surface of the landing area after the touch down (see Figure 7). The height history is closer to a real physical movement. In Figure 9, for horizontal velocity $v_{O,x}$ and $v_{O,y}$, the reconstruction and measurement fit accurately. The extended RTS smoother result gives a bit different estimation of $v_{O,z}$ compared to the GPS measured one. Considering the fact that after the jumper starts gliding down around 610.5 s, the vertical velocity afterward should be positive (moving downwards). So, the vertical speed measured by GPS also contains an error for the in-run part which is associated with the height error. Since the extended RTS smoother considers kinetics ${\dot{h}}_{N}=-v_{O,z}$, the smoother estimated vertical speed is linked to the height estimation. Combining the result in Figure 7 and Figure 8 for the in-run part (around 610.5 s to 616.8 s), the positive $v_{O,z}$ estimates the smoother giving out is more close to the fact that jumper is moving vertically down in the in-run. This indicates that the extended RTS smoother improves the velocity comparing to the GPS with the assistance of information from other sources.

**Figure 10:**
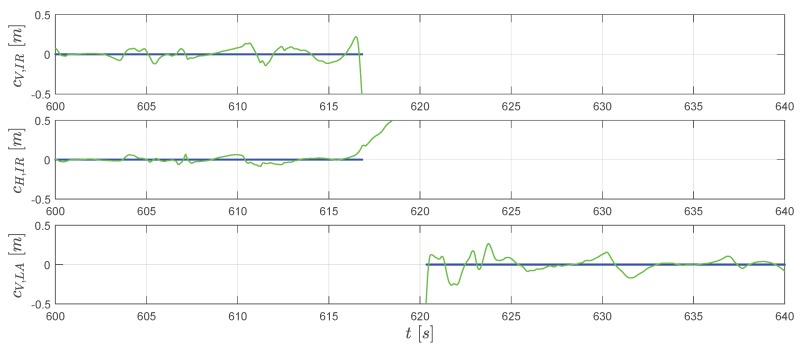
The extended RTS smoother estimated geometric constraints c (green line) and pseudo measurements (blue dots).

The improvement of the estimation accuracy is achieved by fusing data from different sources including measurement sensors, kinematics, and geometric information of the hill. The gyroscope and accelerometer data, after removing the estimated bias error, are used for propagating the states including attitude, velocity, and position together with a priori knowledge of the uncertainties. The GPS measurements and the magnetometer are included as additional data sources for the correction step. Furthermore, the introduction of the geometric constraints from the jumping hill model provides an additional information source, which contributes to the estimation. The constraints $c_{V,\;\mathrm{IR}}$ and $c_{V,\;\mathrm{LA}}$, as shown in Figure 10, establish a strong relationship between states $x_{N}$ and $z_{N}$ on the vertically plane for each part of the trajectory (before take-off and after touchdown).

**Figure 11:**
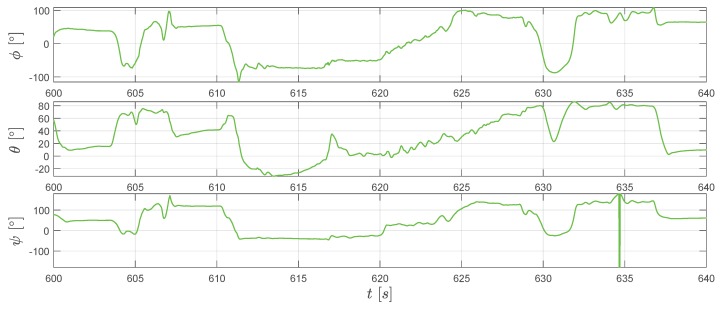
The extended RTS smoother estimated attitude angles: ϕ, θ, and ψ.

**Figure 12:**
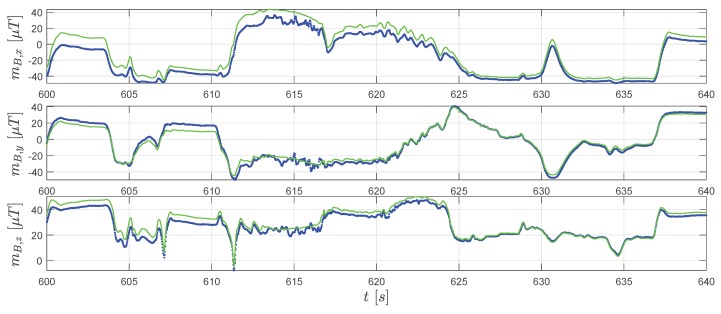
Real magnetometer measurements $m_{B,\;\mathrm{mag}}$ (blue dots) and the extended RTS smoother estimated magnetometer measurements $m_{B,\;\mathrm{meas}}$ (green line).

Although the major aim of applying the extended RTS smoother is to obtain a better position and velocity estimation, it is worth to mention that the RTS smoother also provides an attitude estimation of the IMU sensor. Furthermore, the estimation of the IMU attitude is essential to attribute the accelerometer measured specific forces into the correct direction (see Equations (45) and (49) ). By applying Equations (54)–(56), the converted Euler angles reconstructed by the extended RTS smoother are shown in Figure 11. From Equation (37), we know that the magnetic field measurements change with the attitude. Therefore, the similar tendency of extended RTS smoother estimated $m_{B,\;\mathrm{meas}}$ and magnetometer measurements $m_{B,\mathrm{mag}}$ in Figure 12 indicates good attitude estimation.

**Figure 13:**
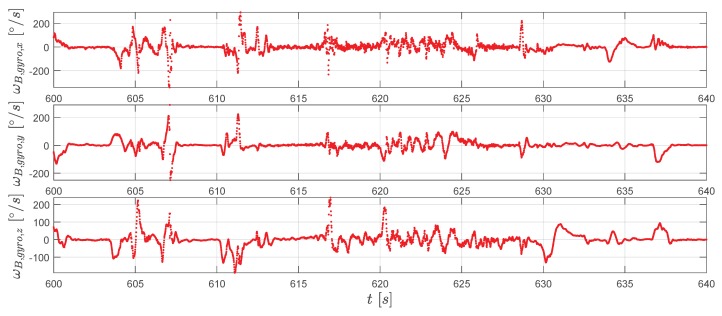
The gyroscope measurements $\omega_{B,\;\mathrm{gyro}}$ (red dots).

**Figure 14:**
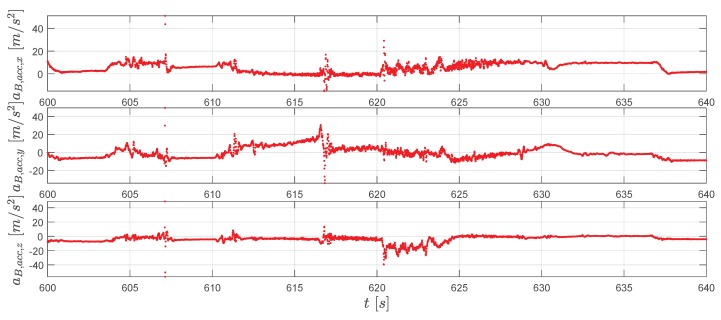
The accelerometer measurements $a_{B,\;\mathrm{acc}}$ (red dots).

To conclude the result presentation, measurements from the gyroscope and the accelerometer are shown in Figure 13 and Figure 14.

**Figure 15:**
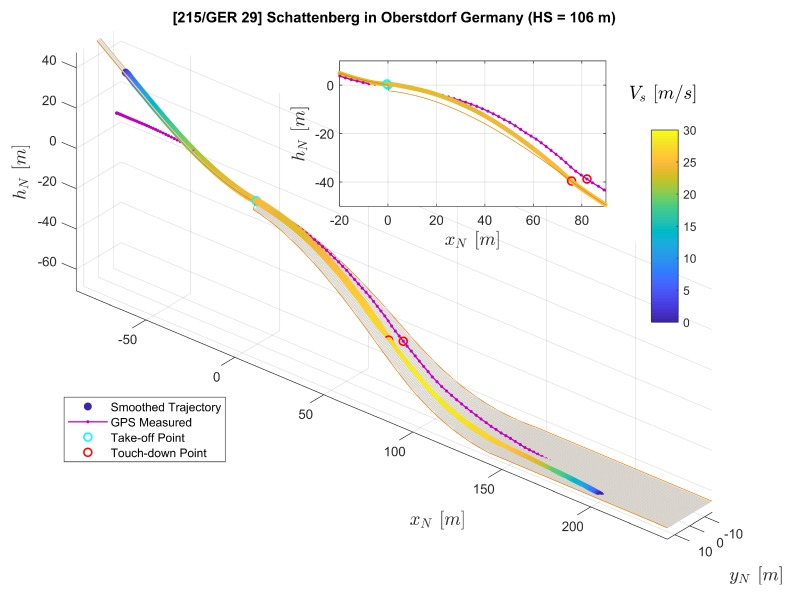
Trajectory Reconstruction result for Jump No. 2.

**Figure 16:**
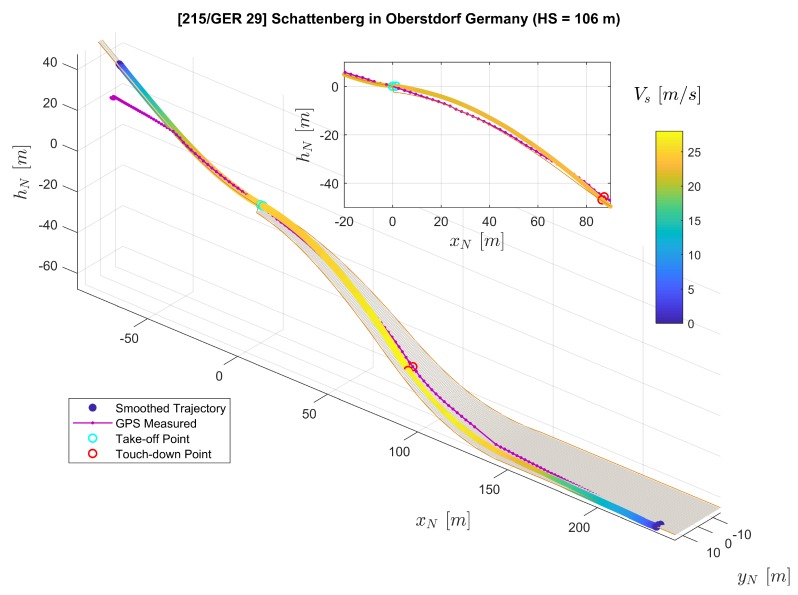
Trajectory Reconstruction result for Jump No. 3.

**Figure 17:**
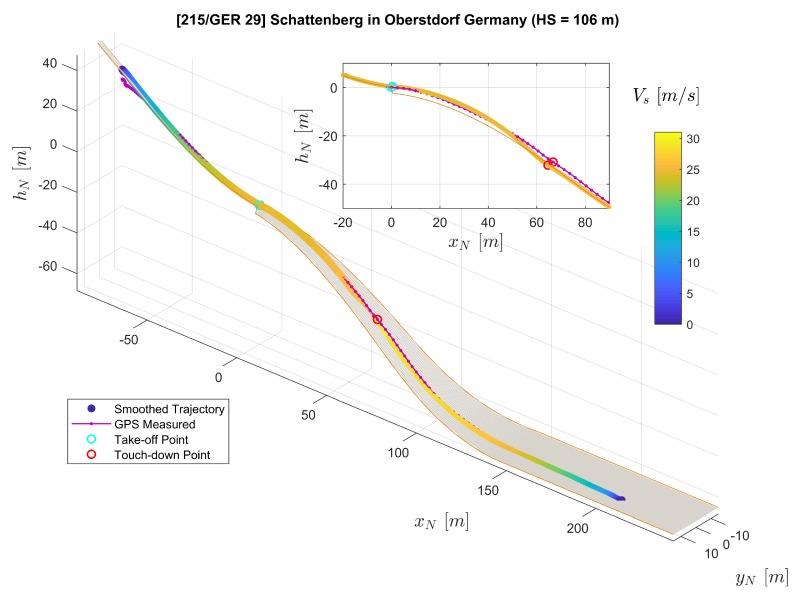
Trajectory Reconstruction result for Jump No. 4.

**Figure 18:**
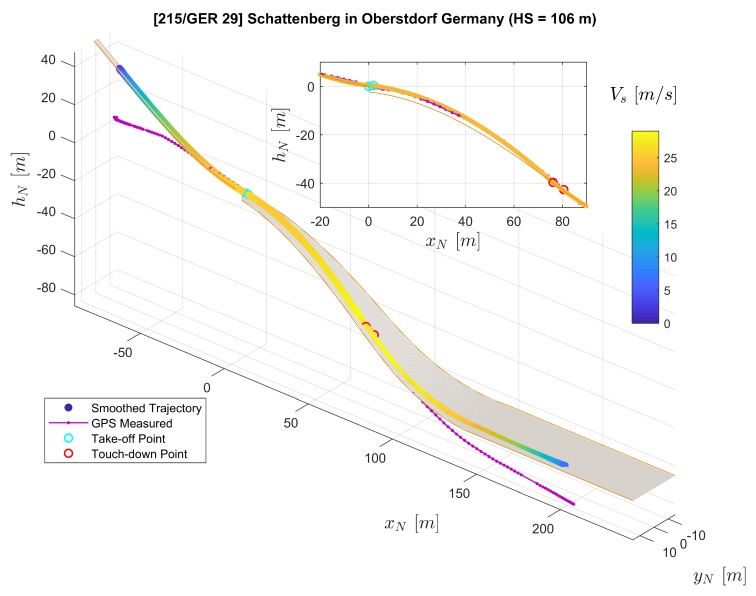
Trajectory Reconstruction result for Jump No. 5.

From the measurement campaign, we collected valid data sets for five different jumps in total. The reconstruction results for the rest of the four jumps are shown in Figure 15, Figure 16, Figure 17 and Figure 18. Comparing to the result from Jump No. 1 (Figure 8), the results show similar characteristics as before which demonstrated the repeatability of the results.

### 4.4. Validation by Jump Length

To validate the trajectory reconstruction results, we calculate the jump length results for the five collected trials obtained from the trajectory reconstruction $L_{\mathrm{TR}}$ for comparison with the jump length measurements from video recordings $L_{\mathrm{VR}}$. The process of determining the jump length from the video recordings is explained in Appendix B. The validation result of five different jumps is shown in Table 7.

The error ΔL in Table 7 is defined as $\Delta L=L_{\mathrm{TR}}-L_{\mathrm{VR}}$, and the RMS error for the jump length estimation is therefore $\bar{\Delta L}=\sqrt{\frac{1}{5}\sum_{i=1}^{5}\Delta L^{2}}=1.0\;m$. Considering that the estimate of the $L_{\mathrm{VR}}$ value could contain an error of 0.5 m as worst case, we calculate the upper bound of the RMS error as ${\bar{\Delta L}}_{\mathrm{ub}}=\sqrt{\frac{1}{5}\sum_{i=1}^{5}{\left(|\Delta L|+0.5\right)}^{2}}=1.5\;m$.

## 5. Conclusions and Discussion

This paper presents a novel method for trajectory reconstruction in ski jumping using wearable sensors by applying the extended RTS smoother with state constraints. The result of simulated measurement data validates the proposed method and shows that it has a theoretical accuracy of decimeter magnitude in positions. The results based on real measurements show the method can successfully reconstruct the trajectories of ski jumps. By formulating the geometric information of the jumping hill as soft equality constraints, the estimation accuracy of positions and velocities is largely improved comparing to the GPS measurements. Jump length data for the five collected trails obtained from video recordings demonstrate good accordance with the reconstruction results with the root-mean-square error upper bound of 1.5m. This provides strong supporting evidence for the validity of the proposed method.

For future research, a quantitative assessment of the accuracy of the proposed method by further real measurement data needs to be carried out. Therefore, another measuring system providing higher accuracy of position and velocity, e.g., a well-calibrated camera system, needs to be employed in order to compare the results of the two systems. For future device developments, wearable sensors providing better position and velocity measurement accuracy, such as differential GPS receivers, which could provide centimeter-level accuracy [6], are recommended to gain better estimation of the jumper’s trajectories. In addition, GPS receivers with higher sampling frequency can be tested which could potentially improve the results. On the other hand, due to the importance of size and weight in the performance of ski jumping, the wearability of such sensors is still a major aspect to be considered. Future measurements should include more trials performed by different athletes in order to increase the statistical confidence. After further development, this technique is promising to be implemented to provide quantified motion feedback in ski jumping training.

## Figures and Tables

**Table 1:** Sensors and their key performance information.

Sensor	Type	Frequency	Performance Characteristics
GPS logger	Qstarz BT Q1000eX	10 Hz	Position accuracy: 3 m circular error probable (50%), velocity accuracy: 0.1 m/s. [13]
IMU	InvenSense ICM-20600	100 Hz	**Gyroscope**: measurement range: $\pm 2000{\;}^{\circ }/$s, rate noise spectral density: $\pm 0.004\;{(}^{\circ }/s)/\sqrt{\mathrm{Hz}}$;
			**Accelerometer**: measurement range: ±16 g, noise spectral density: 100 $\mu g/\sqrt{\mathrm{Hz}}$. [14]
Magnetometer	Alps HSCDTD008A	100 Hz	Measurement range: ±2.4 mT. [15]

**Table 2:** Parameters for the WGS84 reference ellipsoid.

Name	Symbol	Value
The semi-major axis	a	6378137.0m
The first eccentricity	e	0.0818191908426
The meridian radius of curvature	$M_{\mu }\;$	$a\frac{1-e^{2}}{{\left(1-e^{2}\sin^{2}\mu_{0}\right)}^{\frac{3}{2}}}\;$
The radius of curvature in the prime vertical	$N_{\mu }\;$	$\frac{a}{\sqrt{1-e^{2}\sin^{2}\mu_{0}}}\;$

**Table 3:** Diagonal elements of the estimated initial states error covariance matrix ${\bar{P}}_{0}$.

System States	Symbol	Estimated Variance
Position	$r_{N,0}\;$	$\mathrm{diag}{\left(\left[5\;5\;15\right]\right)}^{2}\;\left[m^{2}\right]$
Velocity	$v_{B,0}\;$	$\mathrm{diag}{\left(\left[1\;1\;3\right]\right)}^{2}\;\left[{\left(m/s\right)}^{2}\right]$
Attitude quaternions	$q_{\mathrm{BO},0}\;$	${\left(0.1I_{4}\right)}^{2}\;\left[-\right]$
Gyroscope bias	$\Delta \omega_{0}\;$	${\left(0.0873I_{3}\right)}^{2}\;\left[{\left(\mathrm{rad}/s\right)}^{2}\right]$
Accelerometer bias	$\Delta a_{0}\;$	${\left(0.2I_{3}\right)}^{2}\;\left[{\left({m/s}^{2}\right)}^{2}\right]$
Magnetometer bias	$\Delta m_{0}\;$	${\left(5I_{3}\right)}^{2}\;\left[{\left(\mu T\right)}^{2}\right]$
Magnetometer scaling error	$\Delta S_{m,0}\;$	${\left(0.1I_{3}\right)}^{2}\;\left[-\right]$

**Table 4:** Diagonal elements of the estimated process noise covariance matrix Q.

System Inputs	Symbol	Estimated Variance
Gyroscope	$\omega_{B,\;\mathrm{gyro}}\;$	${\left(0.0175I_{3}\right)}^{2}\;\left[{\left(\mathrm{rad}/s\right)}^{2}\right]$
Accelerometer	$a_{B,\;\mathrm{acc}}\;$	${\left(0.1I_{3}\right)}^{2}\;\left[{\left({m/s}^{2}\right)}^{2}\right]$

**Table 5:** Diagonal elements of the estimated measurement noise covariance matrix R.

System Measurements	Symbol	Estimated Variance
GPS position	$r_{N,\;\mathrm{GPS}}\;$	$\mathrm{diag}{\left(\left[3\;3\;5\right]\right)}^{2}\;\left[m^{2}\right]$
GPS velocity	$v_{O,\;\mathrm{GPS}}\;$	$\mathrm{diag}{\left(\left[0.1\;0.1\;0.3\right]\right)}^{2}\;\left[{\left(m/s\right)}^{2}\right]$
Magnetometer	$m_{B,\;\mathrm{mag}}\;$	${\left(5I_{3}\right)}^{2}\;\left[{\left(\mu T\right)}^{2}\right]$
Quaternions constraint	$c_{q}\;$	$0.01^{2}\;\left[-\right]$
In-run vertical constraint	$c_{V,\;\mathrm{IR}}\;$	$0.1^{2}\;\left[m^{2}\right]$
In-run horizontal constraint	$c_{H,\;\mathrm{IR}}\;$	$0.1^{2}\;\left[m^{2}\right]$
Land area vertical constraint	$c_{V,\;\mathrm{LA}}\;$	$0.1^{2}\;\left[m^{2}\right]$

**Table 6:** The root-mean-square error for estimated variables.

Variables	$\bar{\Delta x_{N}}$	$\bar{\Delta y_{N}}$	$\bar{\Delta z_{N}}$	$\bar{\Delta v_{O,x}}$	$\bar{\Delta v_{O,y}}$	$\bar{\Delta v_{O,z}}$	$\bar{\Delta \phi }$	$\bar{\Delta \theta }$	$\bar{\Delta \psi }$
**RMS Error:**	0.159 m	0.123 m	0.109 m	0.021 m/s	0.026 m/s	0.023 m/s	0.584 ${}^{\circ }$	0.319 ${}^{\circ }$	0.759 ${}^{\circ }$

**Table 7:** Comparison between jump length obtained from the trajectory reconstruction results (proposed method) and video recordings (reference).

Jump Length:	From Trajectory Reconstruction $L_{\mathrm{TR}}$	From Video Recording $L_{\mathrm{VR}}$	Error ΔL
Jump No. 1	91.6 m	90.0 m	1.6 m
Jump No. 2	85.8 m	85.0 m	0.8 m
Jump No. 3	98.6 m	97.5 m	1.1 m
Jump No. 4	69.5 m	70.0 m	-0.5 m
Jump No. 5	86.0 m	85.5 m	0.5 m

**Figure A1:**
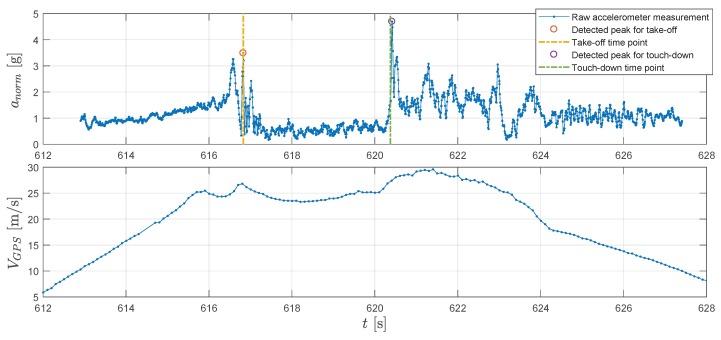
An illustrative example on detecting the take-off and touch-down time points by the raw measurement data.

**Figure A2:**
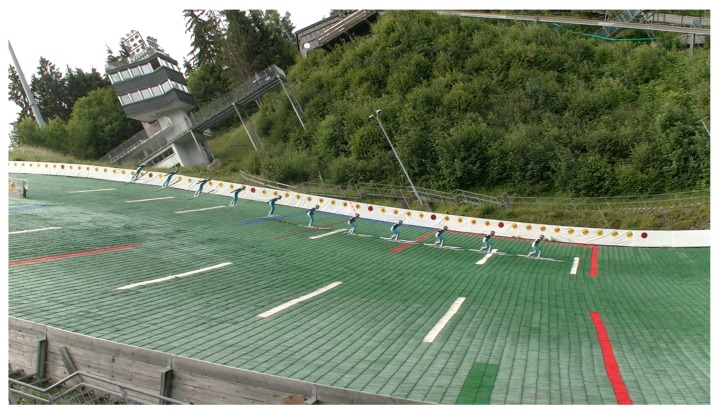
A picture of overlaid snapshots from video recordings to illustrate the jump length detection from video recordings.

## Appendix A. Take-Off and Touch-Down Time Point Detection

The method implemented for detecting the time points for take-off $t_{TO}$ and touch-down $t_{TD}$ is introduced in this section: Due to the change of the ground reaction forces, both take-off and touch-down phase show abrupt changes in acceleration, which is measured by the accelerometer. Previous studies on ski jumping take-off [25] and landing [26,27] also presented similar acceleration changing behavior in their measurement-based results. Therefore, we propose to determine $t_{TO}$ and $t_{TD}$ by detecting these abrupt changes in the accelerometer measurement.

First of all, since the take-off and touchdown would happen when the jumper is moving with a high speed, we limit the analyzing time interval to the part when the GPS measured speed $V_{\mathrm{GPS}}\left(t_{k}\right)={\left|\left|v_{\mathrm{GPS}}\left(t_{k}\right)\right|\right|}_{2}=\sqrt{u_{\mathrm{GPS}}^{2}\left(t_{k}\right)+v_{\mathrm{GPS}}^{2}\left(t_{k}\right)+w_{\mathrm{GPS}}^{2}\left(t_{k}\right)}$ is greater than 10m/s. Next, within this time interval, the L2 norm of the triaxial specific force measured by accelerometer for discrete time points $t_{k}$ are calculated by $a_{\mathrm{norm}}\left(t_{k}\right)={\left|\left|a_{B,\mathrm{acc}}\left(t_{k}\right)\right|\right|}_{2}=\sqrt{a_{x,\;\mathrm{acc}}^{2}\left(t_{k}\right)+a_{y,\;\mathrm{acc}}^{2}\left(t_{k}\right)+a_{z,\;\mathrm{acc}}^{2}\left(t_{k}\right)}$ as shown in Figure A1. Then, two peaks (local maximums) with very large $a_{\mathrm{norm}}$ and time difference more than 1.5s (based on experience) are selected by the following rule as shown in Figure A1 as circles:

(A1) $$\begin{array}{c} t_{peak1}=\underset{t_{k}}{\mathrm{argmax}}\left(a_{\mathrm{norm}}\left(t_{k}\right)\;|\;V_{GPS}\left(t_{k}\right)>10\;m/s\right)\;, \end{array}$$

(A2) $$\begin{array}{c} t_{peak2}=\underset{t_{k}}{\mathrm{argmax}}\left(a_{\mathrm{norm}}\left(t_{k}\right)\;|\;V_{GPS}\left(t_{k}\right)>10\;m/s,\;-1.5\;s\leq t_{k}-t_{peak1}\leq 1.5\;s\right)\;. \end{array}$$

These two peaks indicate large ground reaction forces for take-off and landing. Finally, the take-off time points $t_{TO}$ is determined as the earlier peak. As presented in [26,27], the actual touch-down point in the landing process is a short moment before the maximal ground reaction peak, approximately 0.05s as we estimated. Therefore, the touch-down time points $t_{TD}$ is determined as 0.05s before the later peak as:

(A3) $$\begin{array}{c} t_{TO}=\min\left\{t_{peak1},t_{peak2}\right\}\;, \end{array}$$

(A4) $$\begin{array}{c} t_{TD}=\max\left\{t_{peak1},t_{peak2}\right\}-0.05\;s\;. \end{array}$$

## Appendix B. Jump Length Detected by the Video Recordings

In this section, the process for detecting the jump length detected using the video recordings is explained. Cameras with a frame rate of 50fps are set up at the side of the landing area to record the jumper’s landing movement. Figure A2 shows a picture of overlaid snapshots from one of the video recordings. The time difference between each pair of neighboring snapshots is 0.1s (with a distance of 5 frames). First, the frame for the jumper’s touchdown determined indicated by both skis basically touching the ground. Then, the jump length is detected by the middle point of the ski with the help of the distance markers on the other side of the hill, as shown by the red dash lines in Figure A2. Overall, we consider the detected jump length is within an accuracy of 0.5m.
